# Multimodality Imaging of a Right Atrial Cardiac Mass

**DOI:** 10.7759/cureus.4705

**Published:** 2019-05-21

**Authors:** Manish Kumar, Supriya Tigadi, Michael A Azrin, Agnes S Kim

**Affiliations:** 1 Internal Medicine, University of Connecticut Health Center, Farmington, USA; 2 Cardiology, University of Connecticut Health Center, Farmington, USA

**Keywords:** right atrial mass, melanoma, echocardiography, cardiac mri

## Abstract

Work up of a right atrial mass usually requires multimodality imaging and sometimes a biopsy to affirm histological diagnosis. We present a case of a 74-year-old woman with primary cutaneous melanoma (wildtype BRAF) of the right toe who was found to have a large heterogeneous mass in the right atrium on routine surveillance CT scan. She did not have any cardiac symptoms. Vital signs and physical examination were unremarkable. Cardiac magnetic resonance (CMR) imaging demonstrated a bilobed mass with an intramural component and a mobile blood pool component, with interposed thrombus. Three-dimensional transesophageal echocardiogram (3D-TEE) revealed the mass and its site of attachment on the lateral wall of the right atrium. Given the large size of the tumor and its potential for obstruction of tricuspid inflow, the right atrial mass was surgically resected. Pathology confirmed metastatic melanoma. The patient tolerated cardiac surgery well and was discharged shortly thereafter. In the present case, a large cardiac metastasis was discovered in the absence of clinically detectable disease elsewhere. CMR allowed a comprehensive evaluation of the location, extension, and tissue characterization of the cardiac mass. Transthoracic echocardiogram (TTE) and 3D-TEE allowed assessment of the hemodynamic consequences of this mass and aided in operative planning.

## Introduction

A right atrial mass may be due to a primary or a malignant tumor, thrombus, vegetation or calcification of the tricuspid valve, indwelling catheters, or normal anatomic variants. Early detection of the mass using various noninvasive imaging modalities has important diagnostic and therapeutic implications. Primary cardiac malignancy is rare, however, metastasis is far more common [1]. Most common tumors that metastasize to the heart are lung cancer, breast cancer, and melanoma. Malignant melanoma is well known for its metastatic potential to the heart [2]. We present a case of isolated melanoma metastasizing to the heart in the absence of active disease elsewhere. Multimodality imaging played a pivotal role in the diagnostic workup and management. 

## Case presentation

**Figure 1:**
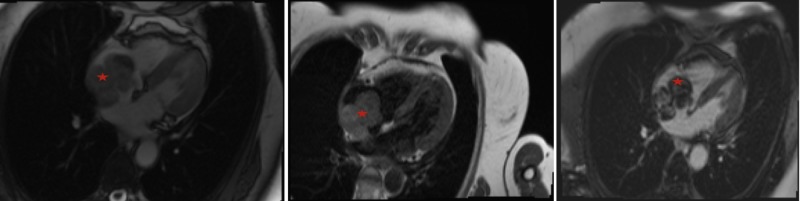
Cardiac MRI imaging of the right atrial mass. Left, SSFP; middle, T1-weighted; right, delayed enhancement images. A bilobed mass (asterisk) was attached to the right atrial wall. The mural component measured 2.3 cm x 4.3 cm x 3.7 cm (TR by AP by cc) and was homogeneously hyperintense to myocardium on T1- and T2-weighted imaging. The mobile, blood pool component of the mass measured 3.1 cm x 4.3 cm x 3.9 cm and was more heterogeneous on T1 than the mural component. The mass demonstrated heterogeneous enhancement on delayed enhancement images. During atrial contraction, the mass appeared to deform the tricuspid valve towards the ventricular cavity.

**Figure 2:**
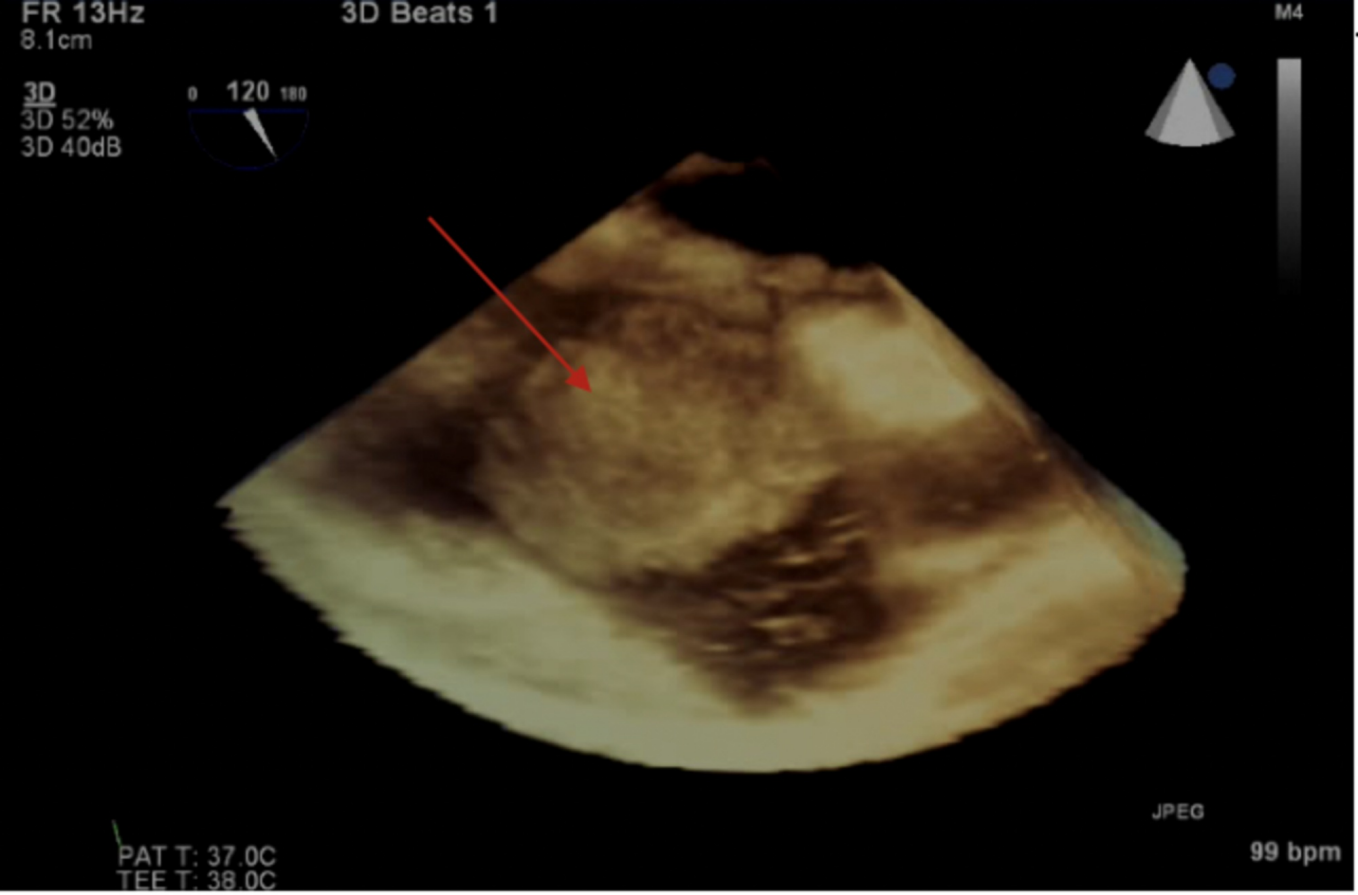
3D TEE showing right atrial mass (arrow). 3D TEE, three-dimensional transesophageal echocardiogram.

A 74-year-old Caucasian female was diagnosed with primary cutaneous melanoma of the right plantar toe in 2014. She underwent surgery followed by an anti-PD-1 agent (Pembrolizumab) immunotherapy resulting in complete resolution. No evidence of metastatic disease at other locations was noted. Three years later, in February 2017, she was found to have a mass in the right ascending colon. She underwent a right hemicolectomy with lymph node dissection and remained disease-free. In May 2017, a CT scan of the chest showed a large heterogeneous mass centered on the lateral wall of the right atrium communicating with another mass inferiorly. She had complaints of slowly progressive fatigue, malaise, and weight loss. Her exam was significant for pallor, increased vitiligo on both hands as well as the forearms and persistent right lower extremity lymphedema. Cardiac magnetic resonance (CMR) (Figure 1) further characterized the mass as a bilobed mass in the right atrium occupying a significant portion of the chamber. One lobe was attached to the right atrial wall with another component that was mobile in the right atrium cavity. The mural component was a solid, enhancing mass. The mobile lobe appeared to be a combination of a mass and thrombus. Both of these masses were homogenously hyperintense to myocardium on T1 and T2 weighted imaging; the mobile component was slightly more heterogeneous on T1 than the mural component. The mass was impinging on the tricuspid valve with the suggestion of tricuspid inflow turbulence and tricuspid regurgitation. The superior vena cava appeared slightly dilated but without involvement. The 3D TEE (Figure 2) revealed the mass and its site of attachment on the lateral wall of the right atrium. Echocardiographic contrast demonstrated enhancement of the mass, indicating vascularity.

The patient underwent TEE-guided biopsy. Two biopsy specimens were obtained and sent for histopathological examination. The procedure was complicated by transient hypotension and development of a small pericardial effusion without echocardiographic features of tamponade. Repeat TTE four days later showed a stable pericardial effusion. Biopsy of the mass was consistent with metastatic melanoma with the tumor being positive for S100, HMB45, and Melan A on immunohistochemistry stain. Given her good functional status and isolated metastasis, she underwent tumor resection from the right atrium with the reconstruction of the right and left atrium, interatrial septum, and left-sided pulmonary veins with bovine pericardial patches. A pacemaker was also placed.

## Discussion

Primary cardiac tumors are rare; however, metastasis is far more common [1]. Breast cancer, lymphoma, and melanoma are some of the common tumors that spread to the heart [2]. Most cardiac metastases are asymptomatic; hence, are frequently diagnosed during surveillance imaging or post-mortem. A high degree of suspicion is required for the diagnosis [2]. Cardiac metastases often involve the right side of the heart. However, the involvement of the left side is also seen [2]. Symptoms when present depend upon the location and size of the mass and may occur due to the involvement of the pericardium, endocardium, or the conduction system.

Echocardiography is usually the initial modality of imaging. TTE is a highly accessible, noninvasive, and inexpensive test modality that allows real-time imaging of intracardiac masses with a high temporal resolution. Various factors, such as obesity and intervening lung tissue can lead to low-resolution images and limit its usefulness [3]. TEE overcomes the limitations of acoustic windows. TEE is more sensitive than TTE for the evaluation of right atrial mass and is useful in further characterizing the shape, size, site of attachment, and associated masses [4]. TEE can also provide imaging of the right atrial appendage, superior and inferior vena cava to rule out a tumor or thrombus located in these structures [3].

The 3D echocardiography yields further clinical information and can aid in preoperative planning as it can describe the spatial information about the mass. It allows simulation of intraoperative visualization of cardiac structures and dynamic surgical anatomy in a real-time manner. Better topographical relationships of structures aid in operative planning [5]. TEE, however, is less sensitive for soft tissue characterization such as myocardial invasion as compared to MRI or contrast-enhanced CT scan. CT scan can assess the heart chambers and surrounding mediastinum, but motion artifact limits its usefulness. Electrocardiogram-gated and contrast-enhanced CT scan overcomes the limitation of motion artifact and provides better sensitivity as compared to conventional CT [6]. MR imaging is, however, superior in detecting tumor infiltration into the myocardium or mediastinal structures [7]. Cardiac metastasis provides a high signal intensity of T1 weighted images due to the presence of melanin and hypointense on T2 weighted images. Not all melanomas have these specific characteristics. Gadolinium enhancement [8] or melanin inversion recovery imaging can further suggest intramural involvement [9]. Recently, positron emission tomography (PET) scan has been used to detect occult or distant metastasis at an early stage [10].

The CT/MRI does not affirm histological confirmation. Biopsy of the intracardiac mass is a high-risk procedure due to dynamic blood flow, cardiac contraction, and highly vascular surrounding structures. Complications of percutaneous biopsy may include tamponade, hemorrhage, sudden cardiovascular collapse, and even death [11]. Various modalities such as open heart surgery, mediastinoscopy, and metastatic mass exploration are the invasive methods that have been used in the past. Less invasive alternative techniques such as biopsy under the guidance of fluoroscopy or transesophageal echocardiography are other alternatives [12]. Recently, transvenous biopsy with intracardiac echocardiography has been used successfully with fewer complications [13].

## Conclusions

Malignant melanoma has the highest predilection for metastasis to the heart. Symptoms are frequently absent, and diagnosis is often delayed. TTE is often the initial modality of choice, and findings can be confirmed with high-resolution images obtained through CMR. CMR allows a comprehensive evaluation of the location, extension, and tissue characterization of the cardiac mass. Melanoma contains melanin, which causes T1 shortening, generating a high signal on T1 images and low signal on T2 images. A biopsy is sometimes required for histological diagnosis, and 3D-TEE can be used to guide percutaneous biopsy.
